# Using machine learning models to improve stroke risk level classification methods of China national stroke screening

**DOI:** 10.1186/s12911-019-0998-2

**Published:** 2019-12-10

**Authors:** Xuemeng Li, Di Bian, Jinghui Yu, Mei Li, Dongsheng Zhao

**Affiliations:** 1 Information Center, Academy of Military Medical Sciences, Beijing, China; 2 School of Electrical and Control Engineering, Xi’an University of Science and Technology|, Xi’an, China; 3 China Stroke Data Center, Beijing, China

**Keywords:** National Stroke Screening, Machine learning models, Risk level classification

## Abstract

**Background:**

With the character of high incidence, high prevalence and high mortality, stroke has brought a heavy burden to families and society in China. In 2009, the Ministry of Health of China launched the China national stroke screening and intervention program, which screens stroke and its risk factors and conducts high-risk population interventions for people aged above 40 years old all over China. In this program, stroke risk factors include hypertension, diabetes, dyslipidemia, smoking, lack of exercise, apparently overweight and family history of stroke. People with more than two risk factors or history of stroke or transient ischemic attack (TIA) are considered as high-risk. However, it is impossible for this criterion to classify stroke risk levels for people with unknown values in fields of risk factors. The missing of stroke risk levels results in reduced efficiency of stroke interventions and inaccuracies in statistical results at the national level. In this paper, we use 2017 national stroke screening data to develop stroke risk classification models based on machine learning algorithms to improve the classification efficiency.

**Method:**

Firstly, we construct training set and test sets and process the imbalance training set based on oversampling and undersampling method. Then, we develop logistic regression model, Naïve Bayesian model, Bayesian network model, decision tree model, neural network model, random forest model, bagged decision tree model, voting model and boosting model with decision trees to classify stroke risk levels.

**Result:**

The recall of the boosting model with decision trees is the highest (99.94%), and the precision of the model based on the random forest is highest (97.33%). Using the random forest model (recall: 98.44%), the recall will be increased by about 2.8% compared with the method currently used, and several thousands more people with high risk of stroke can be identified each year.

**Conclusion:**

Models developed in this paper can improve the current screening method in the way that it can avoid the impact of unknown values, and avoid unnecessary rescreening and intervention expenditures. The national stroke screening program can choose classification models according to the practice need.

## Background

With the character of high incidence, high prevalence, high mortality, high recurrence rate and high disability rate, stroke has become the second most common disease in the world. On the whole, about 13 million patients suffer from stroke in China [1]. Millions of people die of stroke each year in China, and most stroke patients have different degrees of sequelae, which brings a heavy burden to patients and families [2]. In 2009, the Ministry of Health of China launched the China national stroke screening and intervention program and established the China Stroke Data Center [3]. The program established stroke centers which are responsible for screening stroke and intervening its risk factors among residents over 40 years old in China. The China national stroke screening and intervention program conducts the screening every year and conducts follow-up interventions on screened population every 2 years nationwide. Up to now, the program has accumulated nearly 7 million people’s screening data. In the stroke screening program, the risk factors include hypertension, diabetes, atrial fibrillation, dyslipidemia, smoking, apparently overweight or obese, lack of exercise and positive family history of stroke. In the preliminary screening, a person is considered “high-risk” if suffering from more than two risk factors or having a history of stroke or transient ischemic attack (TIA). For those who have been classified to “high-risk” group, further examination (such as computed head tomography and Magnetic Resonance Imaging (MRI) scans) and physician confirmation are needed for intervention suggestion in the rescreening. Population identified as high-risk in rescreening will be followed through telephone every 6 months, and the tests for their blood pressure, blood sugar, and blood lipid are performed every 12 months to make an intervention. The China national stroke screening and intervention program has achieved remarkable results in the prevention and treatment of stroke, and the experience of the past 5 years shows that reasonable intervention for population identified as high-risk can effectively reduce the burden of stroke [4]. Compared with the huge economic burden brought by stroke, expense of rescreening (about 600 Yuan per person or 88 US Dollars per person [4]) is significantly lower.

The screening method currently used in the preliminary screening of the program determines stroke risk levels based on the values of the eight risk factors. However, in the actual screening process, many people are lack of accurate understanding of their own health conditions or not willing to disclose their living habits or health conditions because of some subjective factors. Therefore, some of these risk factors include unknown values, which makes it not possible to determine the stroke risk levels. The missing of stroke risk levels results in reduced efficiency of stroke interventions and inaccuracies in the statistical results at the national level. In original stroke screening data during 2012 to 2017, the total proportion of unknown values in the fields of atrial fibrillation and dyslipidemia exceeds 7%, and the total proportion of unknown values in other factors used to determine stroke levels is also higher than 2%. At the same time, considering the interaction between stroke risk factors, some more fields can be selected as a supplement.

In this study, we use national stroke screening data in 2017 to build machine learning models, aiming to improve stroke risk classification methods currently used in the stroke preliminary screening which cannot avoid effects of unknown values. Accurate classification and reasonable intervention for high-risk population can effectively reduce the burden of stroke on families and the society. It is necessary to consider the recall of the classification model to ensure the pertinence of stroke intervention. Then, we need to ensure that the precision of the model is not too low to reduce unnecessary rescreening and intervention expenditures. Models developed in this paper can be used in the practice of stroke screening program to improve the efficiency of interventions for people with high risk of stroke.

## Method

### Materials

The China national stroke screening and intervention program covers Chinese residents aged above 40 years old in 31 provinces, autonomous regions, municipalities and Xinjiang Production and Construction Corps. In the screening process, a two-stage stratified cluster sampling method is adopted. Firstly, more than 200 screening areas have been selected according to the local population size and total number of counties. Then, an urban community and a township are taken as primary sampling units (PSU) according to the geographical location and local hospital suggestions. In each primary sampling unit, all residents aged over 40 years are surveyed using cluster sampling [5–7]. We take the national stroke screening data in 2017 as the research material. Private information is removed from data by the Stroke Data Center. The national stroke screening data in 2017 includes 747,514 participants after removing participants with error data. Except for those whose stroke risk cannot be classified by the current screening method, participants classified as “high-risk” account for 19.7%. Considering risk factors of stroke may influence each other, we include some more risk factors to provide more information. Besides risk factors defined in the stroke risk classification method currently used, we further include sex, age, drinking history, family history of heart disease, family history of hypertension, family history of diabetes, history of heart disease, heart rhythm and heart murmur as classification features. The definition of features used in models is shown in Additional file 1: Table S1.

### Data pre-processing

The screening data is imbalanced data, and it needs to be pre-processed before the models are established as many machine learning models are sensitive to imbalanced data. Firstly, we use the SMOTE algorithm [8] as oversampling method to increase the amount of data in minority class. The basic idea of the SMOTE algorithm is to analyze samples in minority class and generate new samples based on them. Since it is not simply copying samples from minority class, it can avoid over-fitting to some extent. Then we use undersampling method to randomly sample data in majority class to reduce the difference between amounts of data of the two classes.

We choose participants with stroke risk levels and remove data of people with the history of stroke or TIA. In the preliminary screening, stroke risk levels of three groups of people cannot be judged: people who don’t have known risk factors but have three or more than three unknown risk factors, people who have one known risk factor and have two or more than two unknown risk factors and people who have two known risk factors and have unknown risk factor(s). Data of these three groups of people is also removed from the experimental dataset. We randomly take 70% of the experimental dataset to construct training set, and the remaining 30% of the experimental dataset is used as the test set. Then, we process oversampling and undersampling on the training set.

In 2017 national stroke screening data, the proportion of “unknown” ​​in the field of atrial fibrillation is about 4%, and the proportion of “unknown” in the other fields is about 2%. Participants without stroke risk levels account for about 3%. Inspired by the idea of constructing test sets with occluded areas in testing image recognition models [9], we constructed test sets with missing values in order to simulate the situations that occur in the screening practice. We randomly modified values of risk factors defined by the stroke screening program in test sets to “unknown” according to the above ratio. In test sets, all risk levels of the participants are classified, and the ratio of “unknown” in the field of atrial fibrillation and other fields are about 4 and 2% respectively. Therefore, the test sets can be used to evaluate the classification ability of the models when used to determine the stroke risk levels of patients with missing values.

### Construction of machine learning models

In medical research, common machine learning algorithms for classifying binary results include logistic regression [10], Naïve Bayes [11], Bayesian network [12], decision tree, neural network [13], random forest [14], bagging model, boosting model and voting model. We use Weka package [15] to construct these models to classify stroke risk levels. The grid search method is used to determine which parameter combinations lead to the best performance. When several parameter combinations are optimal and the choice affects the efficiency of the model, we choose parameter combination which leads to the highest efficiency.

We use the C4.5 decision tree algorithm [16, 17] to train and develop decision tree models. We choose the C4.5 decision tree as the sub-classifier of the bagging algorithm (also known as Bootstrap Aggregation algorithm). When the sampling ratio is set as 100%, the bagging algorithm will create a new random sample the same size as the training dataset, but will have a different composition since the sampling process is drawn with replacement, which means that each time an instance is randomly drawn from the training dataset and added to the sample, it is also added back into the training set (replaced). We choose the AdaBoost algorithm to build the boosting model, and choose the C4.5 decision tree as the sub-classifier. The voting model implements several different kinds of sub-classifiers, and votes to obtain the classification result. In the model implementation, we use the logistic regression classifier, naive Bayesian classifier, Bayesian network classifier, decision tree classifier, and neural network classifier as sub-classifiers. The voting method includes the average of sub-classifier results and the majority of sub-classifier results. We choose the average of sub-classifier results as the voting method after testing. Features used in models are shown in Table 1.

**Table 1:** The choice of hyperparameters of each model

Machine learning models	Hyperparameters	Values to be selected	Optimum Value
Decision tree (C4.5)	confidence factor used for pruning (C); minimum number of instances of each leaf (N)	*C* = 0.1,0.15,0.2,0.25, 0.3; *N* = 2,3,4,5,6	*C* = 0.25; *N* = 2
Neural network	the size of network (number of hidden nodes, H); gradient descent (D).	*H* = 3, 4, 8, 10, 20, 50, 100 and *D* = 0.00001, 0.001, 0.1, 0.5, 0.9	*H* = 4; *D* = 0.1
Random forest	the depth of the tree(T); number of tree models(N)	*T* = 1, 2, 3, 5, 10; *N* = 100, 200, 300, 500	*T *= 8; *N* = 300
Bagging with C4.5 decision tree	the sampling ratio (P); number of sub-classifiers(N)	*P* = 70, 80, 90, 95, 100%; *N* = 100, 150, 200, 300, 500	*P* = 90%; *N* = 200
Boosting with C4.5 decision tree	the number of sub-classifiers(N)	*N* = 10, 30, 50, 100	*N* = 30

## Result

After sampling, the training set consists of 408,330 participants, of which 206,164 are labeled as “high-risk”, accounting for 50.5% of the training data. The ratio of each risk factor is shown in Additional file 1: Table S1.

In the experiment, we use the ten-fold cross validation method to evaluate results. We need to consider the recall and precision of models at the same time. The precision is the ratio of the number of truly positive items in the classification result to the number of positive items in the classification result, and the recall is the ratio of the number of truly positive items in the classification result to the number of truly positive items in the entire data set. The F1-score takes into account the precision and recall of the classification model at the same time. AUC refers to area under the ROC (Receiver Operating Characteristic) curve, and it reflects the discriminative ability of models. The formulas for precision, recall and F1-score are as follows. Among them, TP is the number of positive items classified as positive; FP is the number of negative items classified as positive; FN is the number of positive items classified as negative.

1 $$ precision=\frac{TP}{TP+ FP}\ast 100\% $$

2 $$ recall=\frac{TP}{TP+ FN}\ast 100\% $$

3 $$ F1- score=2\ast \frac{precision\ast recall}{precision+ recall} $$

We randomly construct 2000 test sets (with replacement) and calculate averages and 95% confidence intervals of precision, recall, F1-score and AUC using them. Besides the whole test set (test set A), data from test sets that cannot be classified using the current classification method in screening program (test set B) is also used to evaluate the model. Evaluation results of each model are shown in Tables 2 and 3. The bold in tables is the maximum value of that evaluation standard.

**Table 2:** Evaluation results of each model using test set A

Learning method	Precision (95% CI)	Recall (95% CI)	F1-score (95%CI)	AUC (95% CI)
Logistic regression	91.84% [91.81,91.87%]	97.82% [97.76,97.88%]	94.74% [94.69,94.78%]	99.14% [99.09,99.19%]
Naïve Bayesian	69.48% [69.42,69.54%]	97.35% [97.31,97.39%]	81.09% [81.03,81.14%]	98.44% [98.42,98.46%]
Bayesian network	69.66% [69.62,69.70%]	97.55% [97.53,97.57%]	81.28% [81.24,81.31%]	98.41% [98.38,98.44%]
Decision tree(C4.5)	92.25% [92.21,92.29%]	99.83% [99.78,99.88%]	95.89% [95.85,95.94%]	99.92% [99.90,99.94%]
Neural network	92.19% [92.14,92.24%]	99.72% [99.68,99.76%]	95.81% [95.76,95.85%]	99.15% [99.11,99.19%]
Random forest	**97.33% [97.30,97.36%]**	98.44% [98.41,98.47%]	**97.88% [97.85,97.91%]**	**99.94% [99.92,99.96%]**
Bagging with C4.5 decision tree	92.25% [92.22,92.28%]	99.74% [99.71,99.77%]	95.85% [95.82,95.88%]	99.93% [99.92,99.94%]
Voting	94.34% [94.32,94.36%]	99.66% [99.63,99.69%]	96.93% [96.91,96.95%]	**99.94% [99.92,99.96%]**
Boosting with C4.5 decision tree	95.51% [95.48,95.54%]	**99.92% [99.89,99.95%]**	97.67% [97.64,97.70%]	**99.94% [99.91,99.97%]**

*The bold in tables is the maximum value of that evaluation standard

**Table 3:** Evaluation results of each model using test set B

Learning method	Precision (95% CI)	Recall (95% CI)	F1-score (95%CI)	AUC (95% CI)
Logistic regression	31.54% [31.50,31.58%]	94.52% [94.48,94.56%]	47.30% [47.25,47.35%]	71.85% [71.82,71.88%]
Naïve Bayesian	41.98% [41.92,42.04%]	83.44% [83.40,83.48%]	55.86% [55.80,55.92%]	82.37% [82.33,82.41%]
Bayesian network	42.95% [42.91,42.99%]	84.12% [84.08,84.16%]	56.87% [56.82,56.91%]	83.06% [83.04,83.08%]
Decision tree(C4.5)	33.18% [33.14,33.22%]	95.55% [95.51,95.59%]	49.26% [49.21,49.31%]	71.15% [71.12,71.18%]
Neural network	32.72% [32.69,32.75%]	94.86% [94.84,94.88%]	48.66% [48.62,48.69%]	80.33% [80.31,80.35%]
Random forest	**51.34% [51.31,51.37%]**	92.81% [92.78,92.84%]	**66.11% [66.08,66.14%]**	82.52% [82.49,82.55%]
Bagging with C4.5 decision tree	33.06% [33.04,33.08%]	94.57% [94.52,94.62%]	48.99% [48.96,49.02%]	71.02% [70.98,71.06%]
Voting	39.66% [39.62,39.70%]	91.08% [91.03,91.13%]	55.26% [55.21,55.31%]	**85.13% [85.10,85.16%]**
Boosting with C4.5 decision tree	36.35% [36.30,36.40%]	**95.82% [95.79,95.85%]**	52.71% [52.65,52.76%]	80.27% [80.25,80.29%]

*The bold in tables is the maximum value of that evaluation standard

All stroke risk level classification models we developed achieve good performance. The evaluation results show that the recall of the boosting model with decision trees is the highest with both test set A and test set B. And the precision of the random forest model is the highest with both test sets A and test sets B. However, the precision of boosting model with decision tree is lower.

We build the boosting model with decision trees with imbalanced data and balanced data respectively to evaluate the impact of sampling on model results. The recall of the boosting model with decision tree based on imbalanced data is 0.9227(95% CI, 0.9222, 0.9232). The result shows that the recall of the model with balanced data is higher than those with imbalanced data.

We further used national stroke screening data in 2016 as a whole test set to evaluate constructed models, and results are shown in Table 4. Combined results in Tables 2, 3 and 4, models constructed in this paper have good stability. The precision of the random forest is the highest, and the recall of the boosting model with C4.5 decision trees is the highest. The F1-score and AUC of these two models are very close, ranking the top two.

**Table 4:** Evaluation results of each model using screening data in 2016

Learning method	Precision (95% CI)	Recall (95% CI)	F1-score (95%CI)	AUC (95% CI)
Logistic regression	90.56% [90.52,90.60%]	96.35% [96.31,96.39%]	93.37% [93.33,93.41%]	97.96% [99.09,99.19%]
Naïve Bayesian	66.96% [66.93,66.99%]	94.99% [94.95,95.03%]	78.55% [78.51,78.58%]	96.64% [96.62,96.66%]
Bayesian network	67.50% [67.47,67.53%]	93.85% [93.80,93.90%]	78.52% [78.49,78.56%]	96.86% [96.82,96.90%]
Decision tree(C4.5)	91.95% [91.90,92.00%]	98.12% [98.09,98.15%]	94.93% [94.89,94.98%]	99.36% [99.33,99.39%]
Neural network	91.82% [91.78,91.86%]	98.52% [98.49,98.55%]	95.05% [95.02,95.09%]	99.23% [99.20,99.26%]
Random forest	**96.89% [96.86,96.92%]**	95.76% [95.74,95.78%]	96.32% [96.30,96.35%]	**99.41% [99.39,99.43%]**
Bagging with C4.5 decision tree	92.21% [92.19,92.23%]	98.86% [98.83,98.89%]	95.42% [95.39,95.44%]	99.39% [99.92,99.94%]
Voting	92.12% [92.07,92.17%]	98.98% [98.96,99.00%]	95.43% [95.39,95.46%]	99.39% [99.36,99.42%]
Boosting with C4.5 decision tree	94.89% [94.85,94.93%]	**99.12% [99.09,99.15%]**	**96.96% [96.92,96.99%]**	**99.41% [99.38,99.44%]**

*The bold in tables is the maximum value of that evaluation standard

## Discussion

Li X et al. used generalized linear model, Bayes model and decision tree model to predict the risk of ischemic stroke and other thromboembolism of people with atrial fibrillation [18]. Zhang Y et al. employed a variety of filter-based feature selection models to improve the ineffective feature selection in existing research on stroke risk detection [19]. H Asadi et al. applied machine learning to predict the outcome of acute ischemic stroke post intra-arterial therapy [20]. These studies have done good jobs on stroke prediction, but they cannot fully address practical issues raised in the national stroke screening program. Machine learning methods used in this paper are widely used in medical and have achieved good results. Since features don’t satisfy the conditional independence hypothesis in the Naïve Bayesian algorithm and the Bayesian network algorithm, their precision values are lower. Decision tree model, random forest model and neural network model perform well in dealing with fuzzy information. And ensemble learning models can further improve the performance. Austin P C et al. used logistic regression to predict the presence of heart failure with preserved ejection fraction (HFPEF) and proved that it had superior performance [21]. Kaur G et al. used the decision tree model to predict diabetes [22]. Al-Maqaleh B M et al. used decision tree, Naïve Bayesian and neural network to predict the heart disease and compared their performance in term of precision [23]. Jabbar M A et al. developed a random forest model to predict heart disease and its classification accuracy is higher compared to other classification approaches [24]. Based on data from hospital information system, Lee S J et al. used a bagged C4.5 decision tree model to support the medical decision making [25]. Bashir S et al. proposed a bagging model and evaluated it on five different heart disease datasets, four breast cancer datasets, two diabetes datasets, two liver disease datasets and one hepatitis dataset obtained from public repositories [26]. We also used Bayesian network model to study the relationship of risk factors and stroke and found that some stroke prevalence with certain combinations of two risk factors can be higher than that with combinations of three risk factors [27], which can partially solve the problem of missing a few risk factors. And we did not calculate the precision and recall of that model. To the best of our knowledge, there is no model with high recall and precision that can be used to guide stroke risk classification in China national stroke screening and intervention program. Research results of this paper can be used in the practice of the national stroke screening.

Among “high-risk” population in test sets, about 4.36% (95%CI: 4.32–4.40%) of them cannot be identified by the classification method currently used in the stroke preliminary screening, that is, the recall of the current stroke “high-risk” classification method is about 95.64%. All models developed in this paper are better than the stroke “high-risk” classification method currently used in stroke screening program in terms of recall.

There are two usage scenarios for stroke risk classification models developed in this paper corresponding to evaluation results of test sets A and test sets B. The effect of replacing the stroke risk level classification method currently used with the model developed in this paper (scenario1) corresponds to evaluation results using test sets A. In this case, balance between recall and precision should be considered, and we can select models with top two F1-score. For example, the recall of the random forest model reaches 98.44%, which increases the recall of the stroke “high-risk” classification method currently used by about 2.8%. The stroke screening program plans to screen more than one million people every year in next few years in China. Using the random forest model, it is estimated that several thousands more people with high risk of stroke can be identified each year, which may effectively improve the intervention efficiency of the stroke screening program, and will further control the economic burden of stroke in China on individuals, families and the society. At the same time, high precision of the random forest model can reduce unnecessary rescreening and intervention expenditures. If the stroke screening program has more budget and plans to find more residents with high risk levels in the future, the boosting model with decision trees (with highest recall) can be used.

If the model constructed in this paper is used as a supplement to the current screening method to determine the stroke risk levels of the people who cannot be classified by the existing method (about 30,000 people each year, scenario2), the application effect corresponds to evaluation results using test sets B. We should pay attention to the recall of the model in order to identify more people with “high risk” of stroke in this usage scenario. Then the boosting model with decision trees can be used. Its recall reaches 95.82% in this usage scenario, which means that it can successfully identify about 6000^1^ people at “high risk” of stroke who cannot be identified by the current method. At the same time, the precision of the model is about 36.35%, which means that about 15,000^2^ people who are not at “high risk” of stroke are classified as “high-risk” by this model. The classification method currently used can be performed to double check these people for their risk levels before rescreening. Estimation results of this usage scenario are shown in Table 5. In 2016, average hospitalization expenses of cerebral hemorrhage and cerebral infarction patients in China were about 2616 US Dollars and 1380 US Dollars, respectively [4]. Compared with economic burden of stroke, rescreening expenditures are much lower(about 88 US Dollars per person). In the future, we will explore which feature attributes most to classification results of stroke levels.

**Table 5:** Estimation results of supplementing to current screening methods

Learning method	Increased number of identited high-risk people	Number of misidentited high-risk people
Logistic regression	5586	16,492
Naïve Bayesian	4931	13,977
Bayesian network	4971	13,743
Decision tree(C4.5)	5647	16,097
Neural network	5606	16,208
Random forest	5485	11,722
Bagging with C4.5 decision tree	5589	16,126
Voting	5383	14,536
Boosting with C4.5 decision tree	5663	15,333

## Conclusion

In this paper, based on data from China national stroke screening and intervention program in 2017, we build nine models to classify the risk levels of stroke for participants. Models developed in this paper can improve the current screening method in the way that they can avoid the impact of unknown values, and they can improve the efficiency of interventions for people with high risk of stroke while reducing costs for stroke treatment. Models developed can be used in the practice of national stroke screening program.

## Supplementary information

**Additional file 1: Table S1.** The definition of the features used in the model.

## Data Availability

Data that support findings of this study are available from China Stroke Data Center but restrictions are applied to the availability of these data, which were used under license for the current study, and so are not publicly available. Data are however available from authors upon reasonable request and with permission of China Stroke Data Center.

## Abbreviations

AUC: Area Under the ROC curve
BMI: Body Mass Index
CI: Confidence interval
HFPEF: Heart failure with preserved ejection fraction
MRI: Magnetic Resonance Imaging
PSU: Primary sampling units
ROC: Receiver Operating Characteristic
TIA: Transient ischemic attack

## Footnotes

1 1,000,000*3%*19.7%*95.82% ≈ 6000

2 1,000,000*3%* (1–19.7%) * (1–36.35%) ≈ 15,000
