# Supplementary material for: Using machine learning models to improve stroke risk level classification methods of China national stroke screening
Source: BMC Med Inform Decis Mak. 2019 Dec 10;19:261. doi: 10.1186/s12911-019-0998-2 (PMC6902572; doi:10.1186/s12911-019-0998-2)
Supplement: Supplementary file 1 — Additional file 1: Table S1. The definition of the features used in the model. [file 12911_2019_998_MOESM1_ESM.doc]

Table S1 The definition of the features used in the model

| Features | Values | Definition | The ratio in the “high-risk” group (%) | The ratio in the “non-high-risk” group (%) |
| --- | --- | --- | --- | --- |
| Sex | 1 | Male | 48.3 | 49.4 |
|  | 2 | Female | 51.7 | 50.57 |
| Age | 1 | People aged between 40 and 49. | 11.2 | 20.5 |
|  | 2 | People aged between 50 and 59. | 27.6 | 30.7 |
|  | 3 | People aged between 60 and 69. | 36.7 | 30.2 |
|  | 4 | People aged between 70 and 79. | 19.5 | 13.8 |
|  | 5 | People aged above 80. | 5.0 | 4.8 |
| Smoking | 1 | Continuous or cumulative smoking for 6 months, smoking within 30 days prior to the survey, and smoking at least 1 cigarette a day during the survey. | 30.7 | 9.8 |
|  | 0 | Those who do not meet the above criteria for smoking. | 64.2 | 85.4 |
|  | 9 | Unknown | 5.1 | 4.8 |
| Lack of exercise | 1 | Take exercise less than 3 times a week and less than 30 minutes each time for at least one year (those regularly participate in industrial and agricultural activities are excluded). | 53.8 | 20.6 |
|  | 0 | Those who do not meet the above criteria for lack of exercise. | 45.1 | 77.8 |
|  | 9 | Unknown | 1.1 | 1.6 |
| Drinking history | 1 | Those who do not have drinking habits. | 73.9 | 86.2 |
|  | 2 | Consuming large amounts of alcohol frequently (≥3 times/week, ≥100ml each time). | 19.0 | 11.5 |
|  | 3 | Those who rarely drink too much. | 7.0 | 2.3 |
|  | 4 | Those who have had drinking habits but have stopped drinking. | 0.1 | 0.1 |
| Apparently overweight or obese | 1 | BMI(Body Mass Index)≥25 | 36.4 | 6.8 |
|  | 0 | Those who do not meet the above criteria for overweight. | 63.0 | 91.2 |
|  | 9 | Unknown | 0.6 | 3.0 |
| Family history of stroke | 1 | Those whose parents, children or siblings (with same parents) has a history of stroke. | 28.0 | 4.9 |
|  | 0 | People without family history of stroke. | 67.5 | 93.1 |
|  | 9 | Unknown. | 4.5 | 2.0 |
| Family history of heart disease | 1 | Those whose parents, children or siblings (with same parents) has a history of heart disease. | 12.2 | 4.9 |
|  | 0 | People without family history of heart disease. | 84.7 | 92.2 |
|  | 9 | Unknown | 3.1 | 2.8 |
| Family history of hypertension | 1 | Those whose parents, children or siblings (with same parents) has a history of hypertension. | 35.3 | 15.5 |
|  | 0 | People without family history of hypertension. | 61.6 | 81.8 |
|  | 9 | Unknown | 3.1 | 2.8 |
| Family history of diabetes | 1 | Those whose parents, children or siblings (with same parents) has a history of diabetes. | 13.0 | 5.0 |
|  | 0 | People without family history of diabetes. | 83.9 | 92.1 |
|  | 9 | Unknown | 3.2 | 2.9 |
| History of heart disease | 1 | People with coronary heart disease, atrial fibrillation, or valvular heart disease. | 6.6 | 2.1 |
|  | 0 | People without history of heart disease. | 93.0 | 93.6 |
|  | 9 | Unknown | 0.4 | 4.3 |
| Hypertension | 1 | Blood pressure value≥140／90mmHg and systolic pressure≥140mmHg and/or diastolic pressure≥90mmHg without taking anti-hypertensive drugs; has a history of hypertension or takes anti-hypertensive drugs within two weeks (pressure is taken after tranquillization three times in different days). | 85.3 | 30.5 |
|  | 0 | Those who do not meet the above criteria for hypertension. | 11.5 | 66.3 |
|  | 9 | Unknown | 3.2 | 3.2 |
| Diabetes | 1 | Fasting blood-glucose≥7.0 mmol/L; oral glucose tolerance test: 2 hours blood-glucose ≥11.1 mmol/L; Hemoglobin A1C > 6.5%; Random Blood Sugar≥11.1 mmol/L for those with significant hyperglycemia or acute hyperglycemia symptoms; patients who have been diagnosed with diabetes and are being treated are considered to suffer from diabetes | 33.4 | 5.6 |
|  | 0 | Those who do not meet the above criteria for diabetes. | 63.0 | 90.0 |
|  | 9 | Unknown | 3.6 | 4.4 |
| Dyslipidemia | 1 | Glycerin trilaurate≥2.26mmol/L; total cholesterol≥6.22mmol/L; low density lipoprotein cholesterol≥4.14mmol/L; high density lipoprotein cholesterol <1.04 mmol/L. | 74.4 | 27.2 |
|  | 0 | Those who do not meet the above criteria for dyslipidemia. | 23.6 | 70.9 |
|  | 9 | Unknown | 2.0 | 1.9 |
| Atrial fibrillation | 1 | Rapidly disordered fibrillation waves and disappearance of regular atrial electrical activity. (Those with a history of paroxysmal atrial fibrillation should be proved by the diagnosis of hospitals at county level or above.) | 2.1 | 0.2 |
|  | 0 | Those who do not meet the above criteria for atrial fibrillation. | 91.6 | 92.7 |
|  | 9 | Unknown | 6.3 | 7.1 |
| Heart rhythm | 1 | Arrhythmia. | 1.0 | 0.6 |
|  | 0 | People without arrhythmia. | 97.1 | 97.6 |
|  | 9 | Unknown | 2.0 | 1.8 |
| Heart murmur | 1 | Heart murmur. | 94.6 | 96.4 |
|  | 0 | People without heart murmur. | 3.5 | 1.8 |
|  | 9 | Unknown | 2.0 | 1.8 |
